# The nature of alarm communication in *Constrictotermes cyphergaster* (Blattodea: Termitoidea: Termitidae): the integration of chemical and vibroacoustic signals

**DOI:** 10.1242/bio.014084

**Published:** 2015-11-04

**Authors:** Paulo F. Cristaldo, Vojtĕch Jandák, Kateřina Kutalová, Vinícius B. Rodrigues, Marek Brothánek, Ondřej Jiříček, Og DeSouza, Jan Šobotník

**Affiliations:** 1 Laboratório de Interações Ecológicas, Departamento de Ecologia, Universidade Federal de Sergipe, São Cristovão, SE 49000-000, Brazil; 2 Faculty of Electrical Engineering, Czech Technical University in Prague, 166 27 Prague 6, Czech Republic; 3 Faculty of Forestry and Wood Sciences, Czech University of Life Sciences, 165 21 Prague 6–Suchdol, Czech Republic; 4 Faculty of Science, Charles University in Prague, 128 43 Prague 2, Czech Republic; 5 Institute of Organic Chemistry and Biochemistry, Academic of Sciences of the Czech Republic, 166 10 Prague, Czech Republic; 6 Laboratório de Termitologia, Departamento de Entomologia, Universidade Federal de Viçosa, Viçosa, MG 36570-900, Brazil

**Keywords:** Alarm communication, Alarm pheromone, Defence, Isoptera, Nasutitermitinae, Vibroacoustic communication

## Abstract

Alarm signalling is of paramount importance to communication in all social insects. In termites, vibroacoustic and chemical alarm signalling are bound to operate synergistically but have never been studied simultaneously in a single species. Here, we inspected the functional significance of both communication channels in *Constrictotermes cyphergaster* (Termitidae: Nasutitermitinae), confirming the hypothesis that these are not exclusive, but rather complementary processes. In natural situations, the alarm predominantly attracts soldiers, which actively search for the source of a disturbance. Laboratory testing revealed that the frontal gland of soldiers produces a rich mixture of terpenoid compounds including an alarm pheromone. Extensive testing led to identification of the alarm pheromone being composed of abundant monoterpene hydrocarbons (*1S*)-α-pinene and myrcene, along with a minor component, (*E*)-β-ocimene. The vibratory alarm signalling consists of vibratory movements evidenced as bursts; a series of beats produced predominantly by soldiers. Exposing termite groups to various mixtures containing the alarm pheromone (crushed soldier heads, frontal gland extracts, mixture of all monoterpenes, and the alarm pheromone mixture made of standards) resulted in significantly higher activity in the tested groups and also increased intensity of the vibratory alarm communication, with the responses clearly dose-dependent. Lower doses of the pheromone provoked higher numbers of vibratory signals compared to higher doses. Higher doses induced long-term running of all termites without stops necessary to perform vibratory behaviour. Surprisingly, even crushed worker heads led to low (but significant) increases in the alarm responses, suggesting that other unknown compound in the worker's head is perceived and answered by termites. Our results demonstrate the existence of different alarm levels in termites, with lower levels being communicated through vibratory signals, and higher levels causing general alarm or retreat being communicated through the alarm pheromone.

## Abstract (precis)

**Summary:** We inspected the functional significance of both vibroacoustic and chemical communication channels in *Constrictotermes cyphergaster* (Termitidae: Nasutitermitinae), confirming the hypothesis that these are not exclusive but rather complementary processes.

## INTRODUCTION

Predation and competition have long been considered major ecological factors structuring biotic communities (e.g. Shurin and Allen, 2001). In many taxa, a wide range of defensive strategies have evolved helping organisms to deal with costs imposed by such interactions. Strategies consist in combining mechanical (biting, kicking, fleeing) and/or chemical (toxic or repellent compounds) arsenal with an effective alarm communication. The ability to give and perceive conspecific signalling about imminent threats has attained maximum complexity in social groups, such as vertebrates (Manser, 2001, 2013) and insects (Blum, 1985; Hölldobler, 1999; Hölldobler and Wilson, 2009).

In termites, one facet of such complexity is represented by the existence of two forms of alarm transmission: pheromonal and vibroacoustic signalling (Šobotník et al., 2010a). Similarly to other eusocial insects, responses consist of either recruiting nestmates to combat or warning them to hide away (Blum, 1969). Alarm pheromones come from the soldier's frontal gland secretions, and comprise several mono- and sesqui-terpenes (Prestwich, 1984a; Šobotník et al., 2010a). In addition to its alarm function, these secretions also act as defence compounds, being repellent or even toxic to enemies (Prestwich, 1984a). In contrast, vibroacoustic signals consist of vertical and longitudinal oscillatory movements (drumming or shaking, respectively), which are transmitted through substrate-borne vibrations produced when the individual hits the ground and/or ceiling with the head or abdomen (Howse, 1964, 1965; Stuart, 1963, 1988; Röhrig et al., 1999; Hertel et al., 2011). In addition to alarm, vibroacoustic signalling is used in a broad range of behavioural contexts in termites (see Leis et al., 1994; Evans et al., 2005, 2007, 2009) being present in all termite species studied so far (see Howse, 1965; Stuart, 1988; Röhrig et al., 1999; Hertel et al., 2011). Moreover, it is also observed in *Cryptocercus* woodroach, (Bell et al., 2007) a sister group of all termites (Lo et al., 2000; Cameron et al., 2012).

Alarm intricacy in termites is not restricted to the existence of two distinct signals. Both means of alarm signalling can be further enhanced by their very own response. Such a positive feedback occurs in Termitidae: Macrotermitinae (Connétable et al., 1998, 1999; Röhrig et al., 1999), whose soldiers reproduce vibratory signals upon perceiving them from nestmates, thereby amplifying and quickly spreading alarm through the colony. Likewise, in Termitidae: Nasutitermitinae, soldiers expel chemical secretions after being stimulated by the alarm pheromone (Vrkoč et al., 1978; Roisin et al., 1990). Because such secretions contain alarm pheromones, alarm signals are thereby amplified.

Further elaboration in alarm signalling could come from combining these two channels, i.e. conveying alarm through the combination of pheromones and vibroacoustic signalling. To the best of our knowledge, this possibility remains hypothetical: studies on termite alarm have so far focused on one of these two alarm channels, never inspecting their concurrent action. Belonging to the apical Nasutitermitinae subfamily, *Constrictotermes cyphergaster* (Silvestri 1901) termites forages in open-air, with long columns of numerous individuals extending up to hundreds of meters away from the nest (Moura et al., 2006; Bourguignon et al., 2011). Given their foraging ecology, it seems likely that they have experienced enough evolutionary events to develop complex forms of alarm communication. We hypothesize this complexity is achieved by integrating pheromonal and vibroacoustic signaling, and we think that *C. cyphergaster* is a highly suitable organism for testing hypotheses about integrative alarm channels in termites. Thus, in the present study, we investigate and discuss the (i) alarm behavioural responses of *C. cyphergaster* colonies in the field; (ii) anatomical structures involved in chemical alarm propagation, (iii) means of alarm pheromone production, perception and transmission including the behavioural responses of soldiers and workers to alarm pheromones and (iv) biological significance of vibroacoustic alarm communication.

## RESULTS

### Alarm behaviour in the field

In the field, *C. cyphergaster* always showed quick alarm response to disturbance which was experimentally inflicted on the nest wall. The first individual appeared at the nest wall breach, on average, 31±1.8 s (mean±s.e.m.; range from 5 to 60 s) after experimental disturbance was produced. Soldiers were (in 8 out of 10 observations) the first arriving caste, usually followed only by other soldiers; workers appeared in low numbers with a maximum of two (0.3±0.06, mean±s.e.m.) compared to 78 soldiers (26.6±2.44, mean±s.e.m.). Soldiers quickly scattered themselves over the nest surface around the breach, showing typical alarm behaviour, such as running, walking in zigzags, scanning the space with antennae as if searching for signals, performing vibratory signalling, and vigorously touching nestmates.

### Anatomical source of alarm signal

**Fig. 1.**
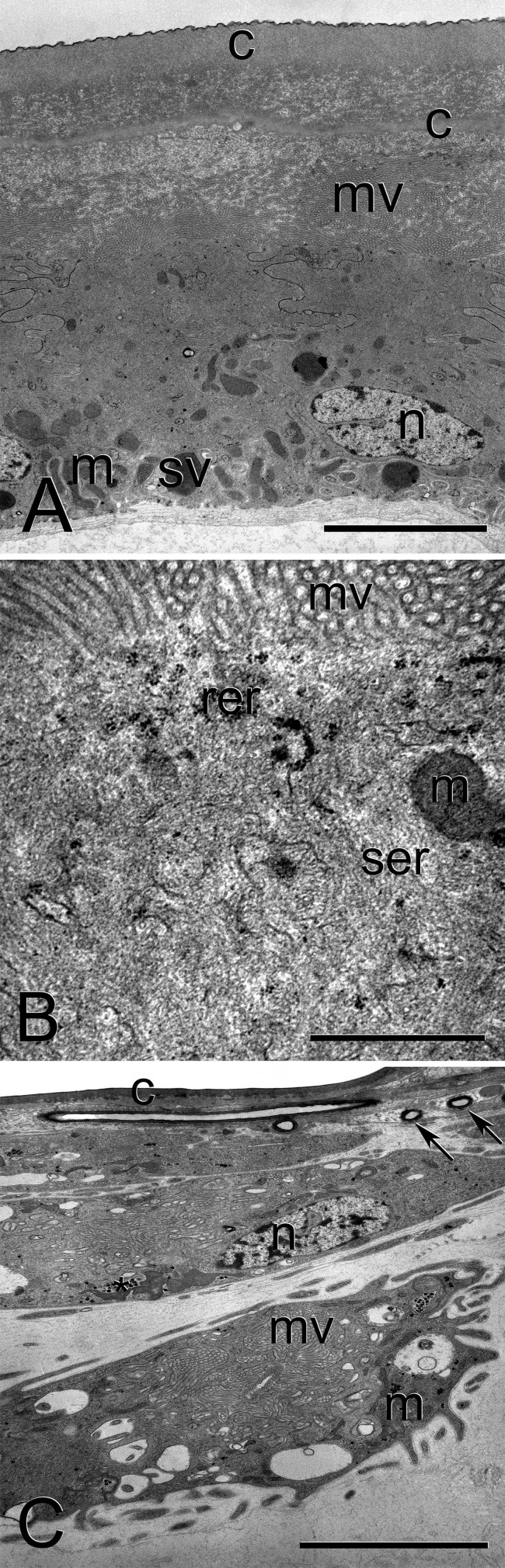
**Ultrastructure of the frontal gland in *Constrictotermes cyphergaster* soldier.** (A) Class 1 secretory cells; note the gap between two layers of cuticle filled with heterogeneous secretion. (B) Detailed view on the apical part of the class 1 secretory cell. (C) Class 3 secretory cells; asterisk marks glycogen rosettes, arrows mark conducting canals. Scale bars: 5 µm (A,C) or 1 µm (B). c, cuticle; m, mitochondria; mv, microvilli; n, nucleus; rer, trough endoplasmic reticulum; ser, smooth endoplasmic reticulum; sv, secretory vesicle.

The frontal gland appeared as a large sac filling a considerable volume of the soldiers’ cephalic capsule, especially dorsal, posterior, and lateral parts. The ventral, posterior, and lateral parts of the frontal gland reservoir were formed by the secretory epithelium, which dorsally and anteriorly changed into non-secretory epithelium to form the evacuating channel, which ended at the tip of nasus. The secretory epithelium of the frontal gland varied in thickness between 5 and 25 µm (in general thicker in the centre, somewhat thinner marginally) and was formed by uniform columnar cells (class 1, *sensu* Noirot and Quennedey, 1974). The epithelium was lined with a highly modified chitin cuticle covered by single-layered epicuticle (Fig. 1A). Populous long microvilli cover the apical parts, while basal parts form shallow invaginations saturating the transport of precursors from the haemolymph. Secretory organelles included a rich tubular smooth endoplasmic reticulum (ER) located predominantly in the apical half of the secretory cells, and rarer rough ER intermingled with smooth ER (Fig. 1B). Secretory vesicles were located predominantly basally, and their content strongly resembled the secretion observed between the cell and the cuticle. Secretory cells class 3 (*sensu* Noirot and Quennedey, 1974) were observed to surround the evacuating channel of the frontal gland and to expel their products into it (Fig. 1C). They were located within the connective tissue (basement membrane formed by multiple layers) surrounding whole frontal gland, but were equipped with own basement membrane of slightly different appearance. The cells were relatively small (about 15 µm in the largest dimension), with relatively large irregular nuclei (up to 8 µm in the largest dimension). Secretory organelles comprise rough ER and numerous Golgi bodies, and produce in concert small electron-dense vesicles dissolve into larger lucent vesicles (up to 2.5 µm in diameter) released into extracellular reservoir into which the evacuating canal is inserted.

### Chemical identity of alarm components

The GC-MS analyses of compounds extracted from frontal gland secretion of *C. cyphergaster* soldiers revealed the presence of seven monoterpene hydrocarbons (Table 1), two sesquiterpene hydrocarbons and a mixture of diterpenes (not shown). Among these, monoterpenes are the most volatile and hence the most likely to compose the alarm pheromone. Behavioural assays conducted on all monoterpenes alone, in pairs, or in triples (see Materials and Methods) revealed that the alarm pheromone of *C. cyphergaster* is mostly likely composed by a combination of (*1S*)-α-pinene, myrcene and (*E*)-β-ocimene (Table 2).

**Table 1.**
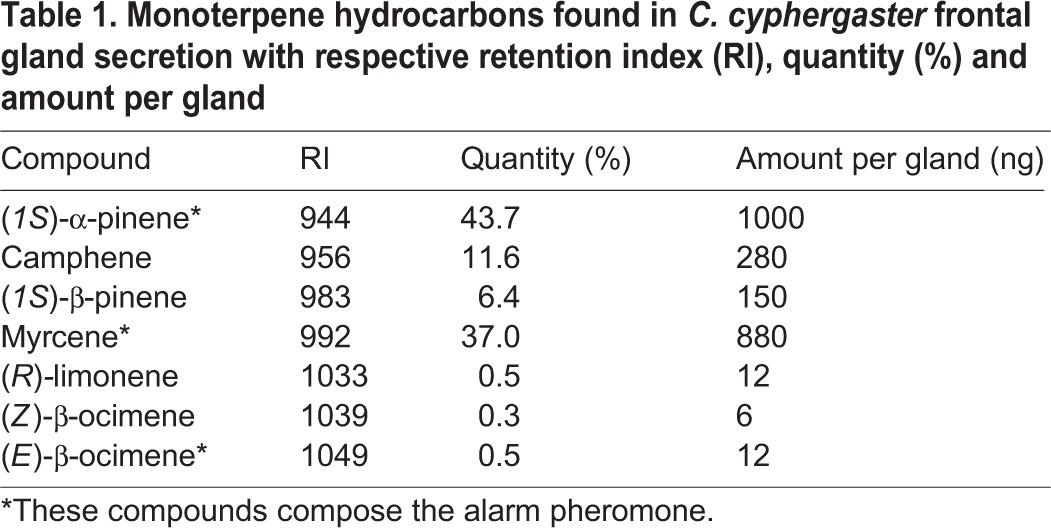
**Monoterpene hydrocarbons found in *C. cyphergaster* frontal gland secretion with respective retention index (RI), quantity (%) and amount per gland**

**Table 2.**
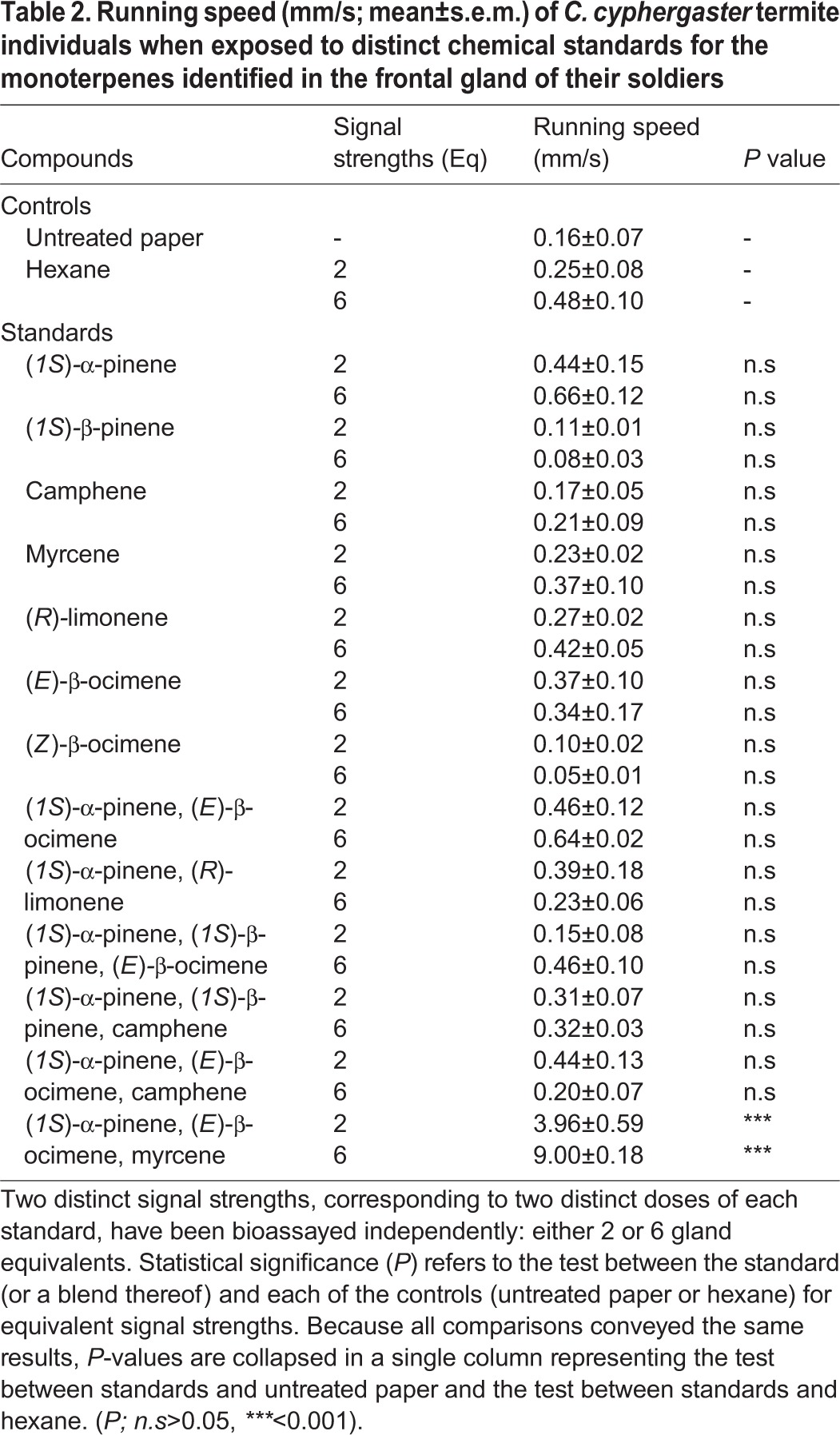
**Running speed (mm/s; mean±s.e.m.) of *C. cyphergaster* termite individuals when exposed to distinct chemical standards for the monoterpenes identified in the frontal gland of their soldiers**

Groups of *C. cyphergaster* increased their running speed significantly only when subjected to the mixture of (*1S*)-α-pinene, myrcene and (*E*)*-*β-ocimene (ANODEV, GLM: *P*<0.001). Thus, this mixture (in natural ratio 100:88:1) is the alarm pheromone (AP) of *C. cyphergaster*.

**Fig. 2.**
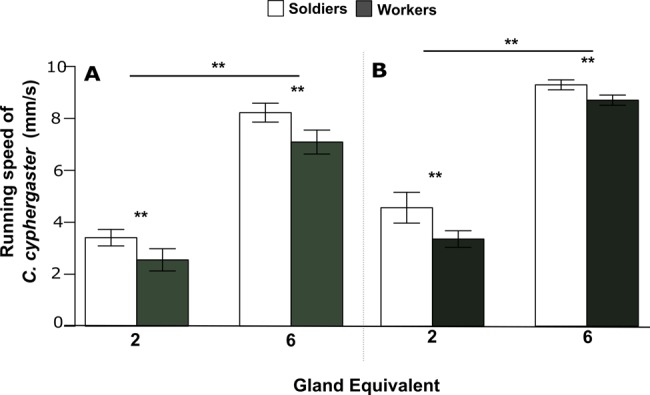
**Effect of synthetic compounds on the running speed of *C. cyphergaster*.** (A) Mixture of all monoterpenes. (B) Alarm pheromone mixture [(*1S*)-α-pinene, (*E*)*-*β-ocimene and myrcene]. Note that although both signal strengths show significant effect, 6 soldier gland equivalent induces higher responses from both soldiers and workers. **P<0.01 (ANODEV, GLM) between responses of castes (soldiers and workers) and gland equivalents (2 and 6). Data represented as mean±s.e.m.

We also tested whether termite groups were sensitive to distinct signal strengths of synthetic compounds [i.e. mixed chemical standards of monoterpenes found in the frontal gland (AM) and AP] showing that the stronger signal (6 soldier glands equivalents) induced adequately stronger responses compared to the lower one (2 soldier gland equivalents), in terms of the running speed of both workers and soldiers (ANODEV, GLM: *P*<0.01; Fig. 2).

### Dose-dependent effects of alarm pheromone on running and shaking movements

**Fig. 3.**
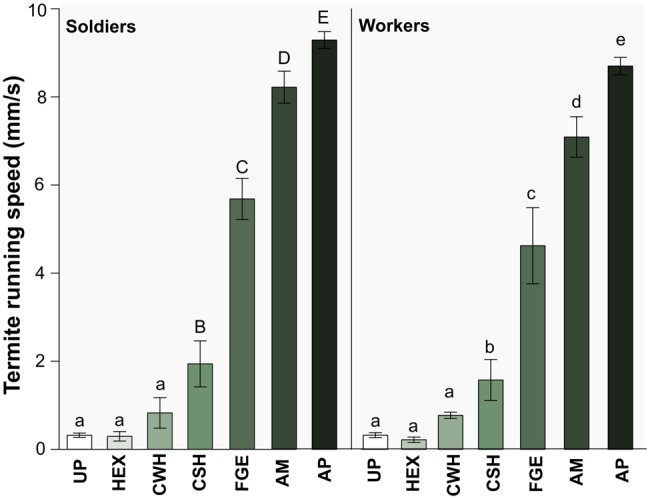
**Running speed of soldiers and workers according to the type of stimulus.** Bars marked with different letters are significantly different (*P*<0.05) within and across panels. Stimuli are arranged in ascending order of alarm pheromone content: from zero pheromones (UP, untreated paper and HEX, hexane) to zero alarm pheromones (CWH, crushed workers head), to increasing purify of alarm pheromones. CSH, crushed soldiers head; FGE, soldier's frontal gland extract; AM, mixture of all monoterpenes found in *C. cyphergaster* frontal gland secretion; AP, alarm pheromone. Data represented as mean±s.e.m.

The running speed of *C. cyphergaster* individuals was strongly affected by different stimuli, with soldiers always moving significantly faster than workers (Likelihood ratio, NLME; *P*<0.0001; Fig. 3). Stimuli were presented as a strip of untreated filter paper (UP) or filter paper loaded with: hexane (HEX), crushed worker heads (CWH), crushed soldier heads (CSH), frontal gland extracts (FGE), mixed chemical standards of monoterpenes found in the frontal gland (AM), pure alarm pheromone (AP) (see Materials and Methods for details). Running responses correlated positively with such stimuli arranged in ascending order according to alarm pheromone dose: from zero pheromones (treatments UP, HEX), to zero alarm pheromones (CWH) to treatments containing alarm pheromones in increased degree of purity (CSH, FGE, AM, AP) (ANODEV, GLM: *P*<0.0001; Fig. 3).

**Fig. 4.**
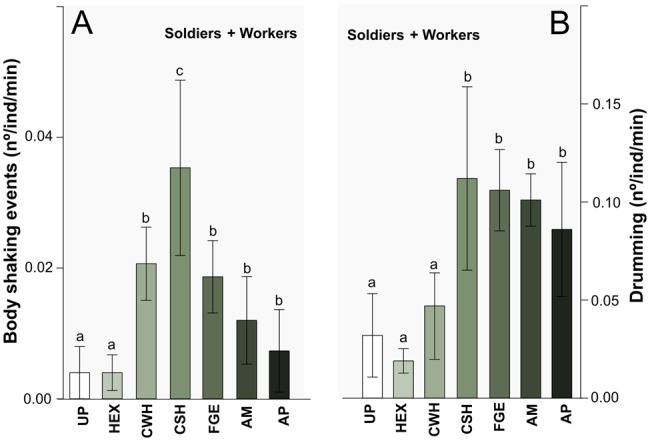
**Shaking and drumming behaviours of soldiers and workers according to the type of stimulus.** Shaking behaviours per individual per minute (A), as scored from video-records bursts (drumming) per minute (B), as scored from vibroacoustic bioassays, in soldiers and workers combined, according to the type of stimulus. The bars marked with different letters are significantly different (*P*<0.04) within panels only. Stimuli are arranged in ascending order of alarm pheromone content: from zero pheromones (UP, untreated paper and HEX, hexane) to zero alarm pheromones (CWH, crushed workers head), to increasing purify of alarm pheromones. CSH, crushed soldiers head; FGE, soldier's frontal gland extract; AM, mixture of all monoterpenes found in *C. cyphergaster* frontal gland secretion; AP, alarm pheromone. Data represented as mean±s.e.m.

The number of body shaking events *per* individual *per* minute, scored from video records, was affected by the stimuli (ANODEV; GLM, *P*=0.04, Fig. 4A). CSH induced the highest numbers of vibratory movements, and CWH, FGE, AM and AP elicit significantly higher numbers of vibratory behaviours than UP and HEX (ANODEV, GLM: *P*<0.04), however, no significant differences among CWH, FGE, AM and AP were detected.

**Fig. 5.**
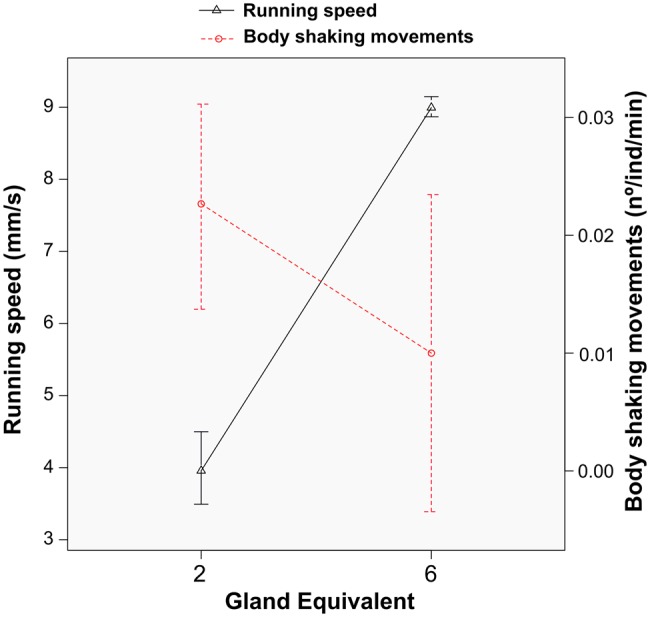
**Dose-dependent effects of alarm pheromone on the running speed (mm/s) and total body vibration of *C. cyphergaster* soldiers and workers during chemical alarm bioassays.** Note that 2 equivalents induced lower running speed o (3.95±0.33) and higher number of drumming behaviours (0.022±0.004), while 6 equivalents provoked higher running speed (8.99±0.16) and lower number of drumming behaviours (0.010±0.0005). Data represented as mean±s.e.m.

**Fig. 6.**
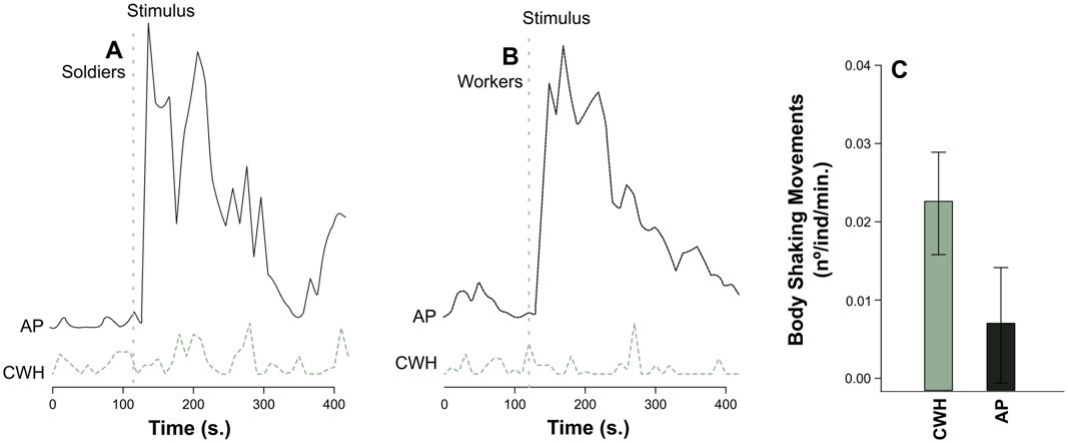
**Comparison of the alarm behaviour dynamics after stimulation by CWH and AP.** The actual running speed of soldiers (A) and workers (B) in the course of the experiment, and the total number of shaking behaviours counted from all recordings (C). The curves represent measured distances travelled by termites second by second. Data represented as mean±s.e.m.

The above evidence that *C. cyphergaster* behavioural responses to alarm pheromones are clearly dose-dependent has been reinforced by subjecting individuals to two distinct doses of alarm pheromones (Fig. 2, Table 3). Moreover, as hinted from previous bioassays (i.e. Fig. 3, Fig. 4A), low doses of AP (2 gland equivalents) induced higher number of body shaking events but lower running speed, while higher doses (6 gland equivalents) triggered a higher running speed but lower number of body shaking events (Fig. 5, Table 3). Thus, termites groups were sensitive to alarm pheromone doses. Increased doses, however, provoked opposite effects on alarm behaviours, increasing running speed and decreasing the level of vibratory communication (see Fig. 6).

**Table 3.**
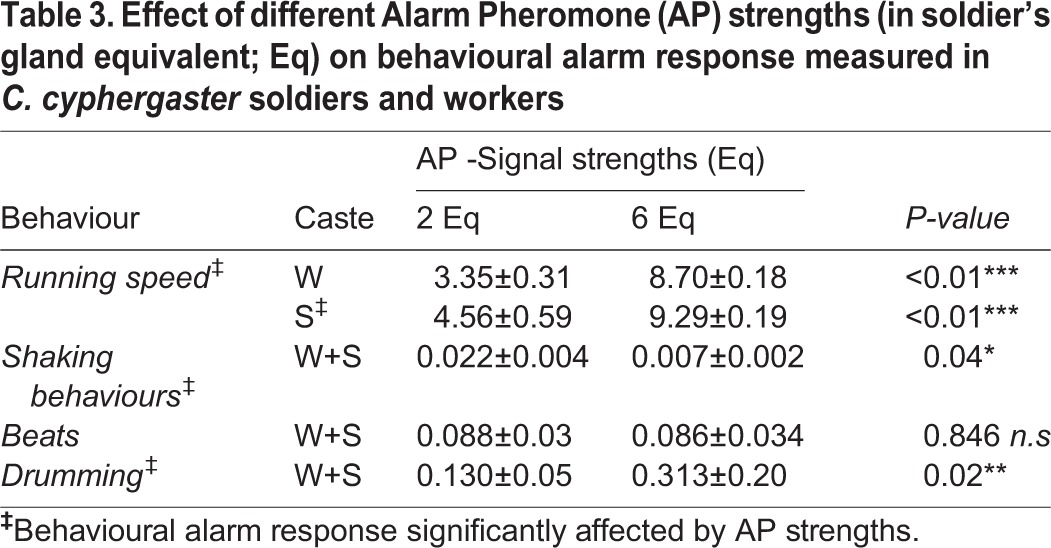
**Effect of different Alarm Pheromone (AP) strengths (in soldier's gland equivalent; Eq) on behavioural alarm response measured in *C. cyphergaster* soldiers and workers**

### Dose-dependent effects of alarm pheromone on vibroacoustic communication

**Fig. 7.**
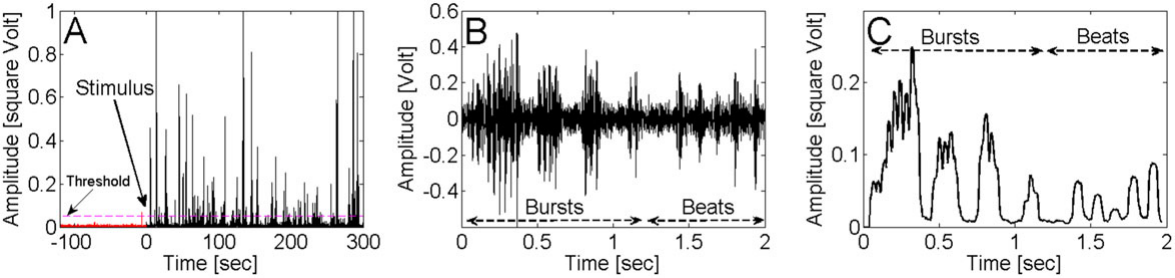
**Physical characteristics of substrate born vibration of *C. cyphergaster* in the vibroacoustic bioassays.** (A) Post-processed response to AP. The threshold (magenta dash line) proportional to power of the vibration signal. Red line represents the activity of the group before insertion the stimulus and black line represents the activity after its insertion. (B) Time domain record of one typical drumming series of *C. cyphergaster*. (C) Post-processed time domain record from figure (B). Note that the series contains four bursts followed by five more singular beats. Burst is a periodic sequence of several beats. Beat is one hit to the ground.

Drumming (an up and down oscillation of the body in a vertical plane; Howse, 1965) is vibratory movements during which the substrate is repeatedly hit by termite body. Because drumming transmits vibration to the substrate it could be recorded by high sensitivity accelerometers in vibroacoustic bioassays. Drumming consisted of solitary beats (corresponding to single hits to the substrate), which were more often combined into bursts of several beats (Fig. 7B). The number of beats per burst ranged from 2 to 7 (3.2±1.2 for soldiers, 2.6±1.1 for workers), and a typical drumming series (see Fig. 7C) included several bursts followed by several more singular beats (Table 4). The number of bursts per minute was affected by the different types of stimuli (ANODEV, GLM, *P*=0.03; Fig. 4B). CSH, FGE, AM and AP (i.e. treatments containing alarm pheromones) increased the number of bursts per minute compared to UP, HEX and CWH (*P*=0.01). On the other hand, the total number of beats per minute was not affected by the different types of stimulus (ANODEV, GLM, *P*=0.90).

**Table 4.**
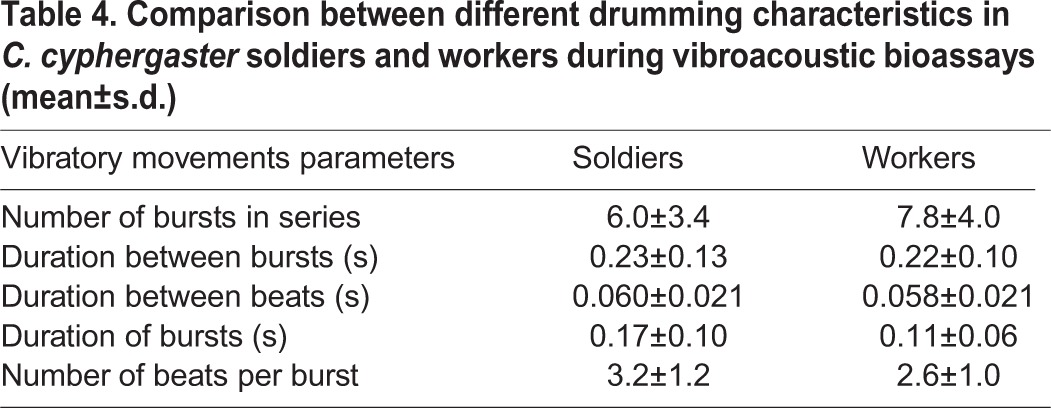
**Comparison between different drumming characteristics in *C. cyphergaster* soldiers and workers during vibroacoustic bioassays (mean±s.d.)**

## DISCUSSION

Nasutitermitinae represent one of the most advanced and successful termite lineages (Bourguignon et al., 2015). As a group, they are cosmotropical and exploit a broad range of diets from sound wood to highly decomposed plant materials in the soil (Donovan et al., 2001; Bourguignon et al., 2011). Only Nasutitermitinae can feed on microepiphytes, a habit which evolved independently and exclusively in the Neotropical genus *Constrictotermes* and several South-East Asian genera (Inward et al., 2007; Bourguignon et al., 2015). This strategy is extremely risky as it exposes vulnerable workers, thus requiring potent defence and efficient alarm communication. Unlike in many other lineages, where soldiers represent rather static mechanical warfare, Nasutitermitinae soldiers are numerous, small and agile, relying exclusively on chemical secretions for efficient defence (Prestwich, 1984a; Šobotník et al., 2010a). Similarly to other Nasutitermitinae, *C. cyphergaster* soldiers eject strings of the defensive secretions from the nasus tip, entangling and incapacitating small enemies, inducing long-term scratching and cleansing behaviour in larger invertebrates, and nauseating even specialised mammal predators (Deligne et al., 1981; Lubin and Montgomery, 1981; Prestwich, 1984a; Šobotník et al., 2010a). At the same time, the frontal gland secretion clearly alerts otherwise calm nestmates, in which it induces profound changes in behaviour towards alarm responses typical of many social insects (Blum, 1981, 1985). These were also described in detail in other termite species and consist of soldiers and workers increasing running speed, gathering at the alarm source or diverting from it, and using vibratory movements to signal emergency (Roisin et al., 1990; Reinhard and Clément, 2002; Reinhard et al., 2003; Šobotník et al., 2008). All above-mentioned behavioural changes were also observed in this study with *C. cyphergaster.* Moreover, we also showed that chemical alarm induces vibratory signalling in *C. cyphergaster* workers and soldiers.

The alarm pheromone originates from the frontal gland, which reveals the same structure as in all other Nasutitermitinae studied so far. The development and structural organization of the frontal gland is indeed one of definitional features of Nasutitermitinae soldiers, along with e.g. the whole soldier head development (see Holmgren, 1909; Noirot, 1969; Santos and Costa-Leonardo, 2006). The fundamental characters of the frontal gland, shared also by *C. cyphergaster*, comprise (i) pear-shaped gland reservoir connected to the fontanelle by rather narrow excretory duct; (ii) posterior, ventral and lateral parts of the reservoir formed by class 1 secretory cells only; (iii) non-secretory epithelium accompanied by secretory cells class 3. On the other hand, our observation excludes the existence of the specific muscle emptying the frontal gland reservoir reported by Santos and Costa-Leonardo (2006). The muscles adjoining the frontal gland reservoir are mandibular muscles, but these lack any connection to the frontal gland reservoir and are only stretched between posterior head (occipital part) and mandibular tendons, similarly to soldiers of all other termites. The combination of reduced mandibles and the presence of well-developed mandibular muscles suggests that the pressure leading to the secretion ejection is indeed created by the mandibular muscles (Holmgren, 1909; Quennedey, 1984), but without the direct junction between the muscles and the frontal gland reservoir. The tentorial-fontanellar muscle, observed in *Nasutitermes* by Noirot (1969), indeed occurs also in *C. cyphergaster*, and may prevent fontanelle from being plugged up by the frontal gland epithelium under increased pressure, as already discussed by Šobotník et al. (2010b) and Kutalová et al. (2013).

The frontal gland secretion of Nasutitermitinae soldiers is made of sticky diterpenes dissolved in a monoterpene mixture (for review see Prestwich, 1984a or Šobotník et al., 2010a). While the function of irritating agents can be a commonplace for all monoterpenes (Deligne et al., 1981; Baker and Walmsley, 1982; Prestwich, 1984a), the alarm pheromone function was proved only in a few monoterpenes produced by *Nasutitermes* (Vrkoč et al., 1978; Roisin et al., 1990) and *Velocitermes* (Valterová et al., 1988) soldiers. α-pinene is the most common compound showing the alarm pheromone function, occurring alone in *N. princeps* (Roisin et al., 1990) or in mixture in *N. ripertii, N. costalis* (Vrkoč et al., 1978), *Velocitermes velox* (Valterová et al., 1988) and *C. cyphergaster* (present study). Surprisingly, in our case this compound shows no activity by itself, but is essential for the blend function (Table 2). Two other components responsible for the chemical alarm signal in *C. cyphergaster* are (*E*)*-*β-ocimene and myrcene, both matched to the pheromone function for the first time in termites. While (*1S*)-α-pinene and myrcene represent dominant components of *C. cyphergaster* frontal gland secretion*,* (*E*)*-*β-ocimene represents only a minor component, but which is still needed for the alarm function of the secretion (see Table 2). (*E*)*-*β-ocimene is known to act as an aggregation pheromone in darkling beetle *Alphitobius diaperinus* (Bartelt et al., 2009) and is also present in the venom gland secretions of several ants and wasps (Keegans et al., 1993; Dani et al., 1998). Myrcene is a common compound produced by termite frontal glands (Šobotník et al., 2010a), but is also known as the sex pheromone in shield bugs (Pentatomidae: Acanthosomatinae; Staddon, 1990) and some click beetles (Elateridae: *Agriotes*; Yatsynin et al., 1996). All other monoterpenes identified in *C. cyphergaster* frontal gland secretion are known to occur in other Nasutitermitinae species, except for (*E*)*-*β-ocimene and (*Z*)*-*β-ocimene found only in *Syntermes* (Syntermitinae) (Baker et al., 1981). Different classes of compounds (alcohols, aromatic compounds and a ketone) were identified by Azevedo et al. (2006), in addition to monoterpenes partially overlapping with our results. Such differences (distinct chemotypes) are repeatedly reported in termites, especially due to geographic distance (e.g. Goh et al., 1984; Prestwich, 1984b; Valterová et al., 1989; Krasulová et al., 2012), and evolutionary history (Quintana et al., 2003; Perdereau et al., 2010; Krasulová et al., 2012). Larger molecules were out from the scope of our work, but several sesquiterpenes and a diterpene were previously identified in *C. cyphergaster* soldier frontal gland secretion as well (see Baker et al., 1984; Azevedo et al., 2006). The other case of alarm pheromone presence in termites occurred in *Mastotermes*, but in contrary to Neoisoptera, the pheromone is benzoquinone produced by the labial glands (Delattre et al., 2015).

The other component of termite alarm signalling, the vibroacoustic communication by means of substrate-borne vibrations, occurs in all termite species studied so far (see Hager and Kirchner, 2013; Howse, 1962, 1964, 1965; Stuart, 1963, 1988; Kirchner et al., 1994; Connétable et al., 1998, 1999; Röhrig et al., 1999; Reinhard and Clément, 2002; Hertel et al., 2011; Delattre et al., 2015), as well as in *Cryptocercus* (Bell et al., 2007), the sister-group of all termites (Lo et al., 2000; Cameron et al., 2012). Although other cockroaches are able to perceive the substrate-borne vibration (Bell et al., 2007), the communication through drumming is an evolutionary novelty of *Cryptocercus*-Termitoidea clade. Our results showed that the vibratory communication is used also in meanings different from the alarm, as only the number of bursts (drumming series; Fig. 4B) significantly increased according to the type of stimulus, while the total number of beats did not. These results may indicate that termites communicate with nestmates using singular beats and the disturbance induces only a change in the vibratory communication patterns. Bursts of beats are indeed known to bear alarm function in termites (Kirchner et al., 1994; Connétable et al., 1998, 1999; Röhrig et al., 1999; Reinhard and Clément, 2002; Hertel et al., 2011), but as the total number of beats per experiment was never scored before, our results cannot be compared with any other reports. At the same time, the vibrations can also be used for other purposes, such as assessing the remaining wood size and quality (Evans et al., 2005, 2007; Inta et al., 2007) or the presence of competitors (Evans et al., 2009). In fact, as noted long ago, vibratory communication is virtually omnipresent in termite colonies and the rich tactile communication is mostly based on vibratory signals (Sbrenna et al., 1992; Leis et al., 1994).

The most surprising finding is the significant increase in the number of shaking behaviours in response to CSH, followed by a mild, significantly lower increase provoked by all other termite-related treatments (CWH, FGE, AM, AP; see Fig. 4A). CSH enhanced the total number of body shaking behaviours more than FGE, AM, AP and CWH. Moreover, shaking response elicited by the latter (CWH) did not differ from the shaking response elicited by the other treatments containing alarm pheromone (including AP, i.e. pure alarm pheromone). That this behaviour was affected by some compound(s) of termite origin is confirmed by the fact that blank controls (UP and HEX) did differ from termites-related treatments in terms of shaking behaviours. At the same time, running speed was not increased by CWH as compared with blank controls, but did depend on the alarm pheromone treatments (Figs 3, 6), the same happening with drumming (Fig. 4B). In summary, while running and drumming were elicited by alarm pheromones, shaking was elicited by both alarm pheromone and some additional compound(s) present in the workers' heads. These compounds seem be present too in the soldiers' heads and act in concert with alarm pheromone in eliciting shaking movements. These results point at two interesting phenomena:

(A) Some compounds present in heads of both worker and soldier are perceived by other colony members, which respond to them by shaking behaviour but neither by increased running speed nor by drumming. These cues may originate e.g. from mandibular glands, which occur in Nasutitermitinae soldiers (Noirot, 1969) and are considerably enlarged in *C. cyphergaster* workers (Costa-Leonardo and Shields, 1990). At the same time, these compounds may also originate from the frontal gland, which also occurs in *C. cyphergaster* workers (J.Š., unpublished) and is developed similarly to soldierless Apicotermitinae (Šobotník et al., 2010c). The repellent effect of whole worker body extracts was observed to affect *C. cyphergaster* trail-following behaviour (Cristaldo et al., 2014), and the compounds responsible for it can actually be the same.

(B) There are different levels of excitement, and these are communicated by increasing vigorousness of vibratory behaviour (and amounts of released alarm pheromone; see Vrkoč et al., 1978), of which only the most intense can be detected by accelerometers. The combined observations suggest that the vibration intensity is indeed scaled from shaking to drumming and the lower intensities serve only for short-range or tactile communication, which represent instead a warning signal. In fact, our results show that the alarm behaviour includes more components, which are mediated by different means and are displayed by different intensities probably according to disturbance nature and intensity (‘dose-dependent effect’; see Fig. 5). For example, the AP at 2 equivalents provoked a lower running speed and drumming but a high number of shaking behaviours, while 6 equivalents induced a high running speed, drumming series and lower numbers of shakings. One can easily imagine the natural significance of different alarm levels as a proper response to differing threats. While the proper response to, e.g. a wasp digging in the nest wall (J.Š., personal observation), is to fight for the nest integrity, an anteater attack should better be responded by a mass retreat of vulnerable stages and recruitment of all available soldiers. After all, insectivores tolerate rather lower doses of soldiers’ defensive secretions (Lubin and Montgomery, 1981).

To conclude, termites are eusocial cockroaches (Lo et al., 2000; Inward et al., 2007; Cameron et al., 2012), and their complex social life made them use more sophisticated means of communication compared to their solitary relatives. Concerning the alarm function, termites and *Cryptocercus* evolved alarm communication using body vibrations, and the use of alarm pheromones evolved twice among termites, in *Mastotermes* (Delattre et al., 2015) and in Neoisoptera (present study). Our results demonstrate the dual function of the alarm signalling in *C. cyphergaster*: In addition to vibratory communication occurring in all termites, the soldier frontal gland secretion contains the alarm pheromone, which is a mixture of abundant [(*1S*)-α-pinene, myrcene] and rare [(*E*)-β-ocimene] monoterpenes, all contributing to the desired function. The three-component alarm pheromone is reported for the first time in termites, and may provide necessary specificity to alarm signals even in the open air, where *C. cyphergaster* is foraging for food. The behavioural changes due to the chemical alarm signalling include the increase of the running speed and the number of vibratory movements in both soldiers and workers (Movie 1). The responses are clearly dose-dependent, as lower doses (represented here by 2 soldier equivalents) enhance more the vibratory communication while higher doses (represented by 6 soldier equivalents) more strongly affect the running activity.

## MATERIAL AND METHODS

### Termites and ethical note

Colonies of *C. cyphergaster* were collected near Sete Lagoas town (S 27°19′, W 14°44′; Minas Gerais, Brazil). A total of 30 colonies were used for all chemical analysis and behavioural assays. Of the 30 colonies, 10 were biossayed in the field to find evidence of alarm behaviour, six were bioassayed in the lab to identify monoterpenes most active in alarm, six were bioassayed using video recordings in the lab to inspect termite responses to chemical elicitors, six were bioassayed using an accelerometer to inspect termite vibroacoustic responses to these same chemical elicitors, two were used for anatomical studies. Colonies were collected in July or September 2012 and immediately transported to Viçosa (Minas Gerais, Brazil) or Prague (Czech Republic). In both places, termites were kept in the laboratory under controlled conditions (±27°C and low relative air humidity). Termite collections in Brazil were carried out with permission of IBAMA (33094) to ODS, PFC and JŠ, permission from EMBRAPA (The Brazilian Enterprise for Agricultural Research) to conduct the study on their site, as well as tacit approval from the Brazilian Federal Government implied by formally granting ODS the post of Scientific Researcher. Material transport was in agreement with Brazilian and Czech laws; export permits from Brazil was provided by CNPq-Brazil (001347/2012-8) and import permits into Czech Republic, by Division of Protection against Harmful Organism of the Czech Republic (SRS 032901/2012 and 032904/2012).

Field assays took place in July 2012 in Sete Lagoas. Part of lab bioassays took place in Sete Lagoas and Viçosa MG Brazil, in July 2012. Considerable parts of colonies collected in September 2012 were transported to Prague (Czech Republic), and kept inside plastic boxes at about 27°C and low relative humidity. Anatomical and chemical characterization of frontal gland extracts, behavioural assays to test alarm responses to particular chemical standards, and vibrational recordings were done in Prague. All assays are fully described below.

### Alarm behaviour in the field

In order to observe the alarm behaviour in natural conditions, 10 arboreal nests of *C. cyphergaster* were subjected to experimental disturbance using the blade of a pocket knife to jab the nest on its exterior wall, forming a cylindrical hole (approx. ø 1 cm × 3 cm long) midway between the nest's base and top. A wooden stick c.a. 20 cm long was immediately and partially inserted into this hole, left for 40 s after which clinging worker and soldier termites were shaken into a plastic tray held right below the stick. These termites were then quantified. Behaviour of termites up-surging at this breach was noted concomitantly.

### Anatomical source of alarm signal

The structural analysis was carried out through optical and electron microscopy in order to find evidence for the functional role of the frontal gland as source or merely storage of its secretions. Ten *C. cyphergaster* soldiers were anaesthetized on ice and fixed according to protocols described in Šobotník et al. (2010b,c). Semithin sections (0.5 µm) were cut using Reichert-Jung Ultracut Microtome and stained with methylene blue for optical microscopy. Sections were studied using a Carl-Zeiss Amplival optical microscope combined with Canon EOS 500D digital camera. For transmission electron microscopy, ultrathin sections (60-80 µm) were stained according to Reynolds’ protocol (Reynolds, 1963) and observed using Jeol 1011 transmission electron microscope.

### Chemical identity of alarm components

Gas chromatography (GC) coupled with mass spectrometric (MS) detector (quadrupole DSQ II, Thermo Scientific) was used to study composition of the extracts. The nonpolar ZB-5MS column (30 min, id 0.25 mm, 0.25 μm phase thickness) was used. Temperature program was 50°C to 120°C at 8°C/min rate and then to 320°C at 15°C/min rate. 1 μl of the extracts (representing approximately 1/10 of termite equivalent) was injected in a splitless mode. Helium was used as a carrier gas at a constant flow rate of 1 ml/min. Identification of particular compounds was based on a comparison of their retention indices and fragmentation patterns with MS library (NIST MS Search 2.0) and the published data (Adams, 2007). The retention indices were calculated from the retention times of n-alkanes (nC10-nC30).

To distinguish between enantiomers of detected monoterpenes we used GC-FID (HP 6850 Series) with chiral column HP-CHIRAL-20B (30 min, id 0.25 mm, 0.25 μm phase thickness). Temperature program was from 40°C (1 min) to 60°C (13 min) at the rate of 50°C/min then to 110°C (12 min) at 10°C/min and finally at the same rate to 150°C (50 min). Hydrogen was used as a carrier gas. The final identity of the compounds and the enantiomers was again established based on comparison with the standards. The identification of particular compounds (including stereochemistry) and their quantification was possible thanks to possession of all standards in IOCB laboratory store or their purchasing from a company.

Each of the extracts were prepared from 10 soldier heads (nasus cut off) submerged into hexane (10 µl per head), macerating overnight at 4°C followed by another washing with 5-10 μl of hexane per head. Final samples were stored at −18°C and used for chemical analyses and behavioural bioassays, in which we always worked with recounted soldier equivalents (1 individual extracted in known solvent volume).

In order to determine the combination of monoterpenes more likely to act as alarm pheromone, bioassays were conducted presenting termites with filter paper impregnated with chemical standards for the monoterpenes detected by GC-MS in the frontal gland extracts. Bioassays consisted of digitally video-recording termites confined in a Petri dish to estimate their running speed, as described below. Such termites were submitted to (i) a given monoterpene alone, (ii) a given pair among all possible pairs of monoterpenes, and (iii) a given triple among all possible triples of monoterpenes as long as they are formed by one pair previously detected as behaviourally active. A given bioassay corresponding to a given combination of monoterpenes was repeated six times, each time using a single Petri dish with termites from a single colony. Each group was tested twice, once for control and other time for termite-related sample, in random order.

### Dose-dependent effects of alarm pheromone on running and shaking movements

**Fig. 8.**
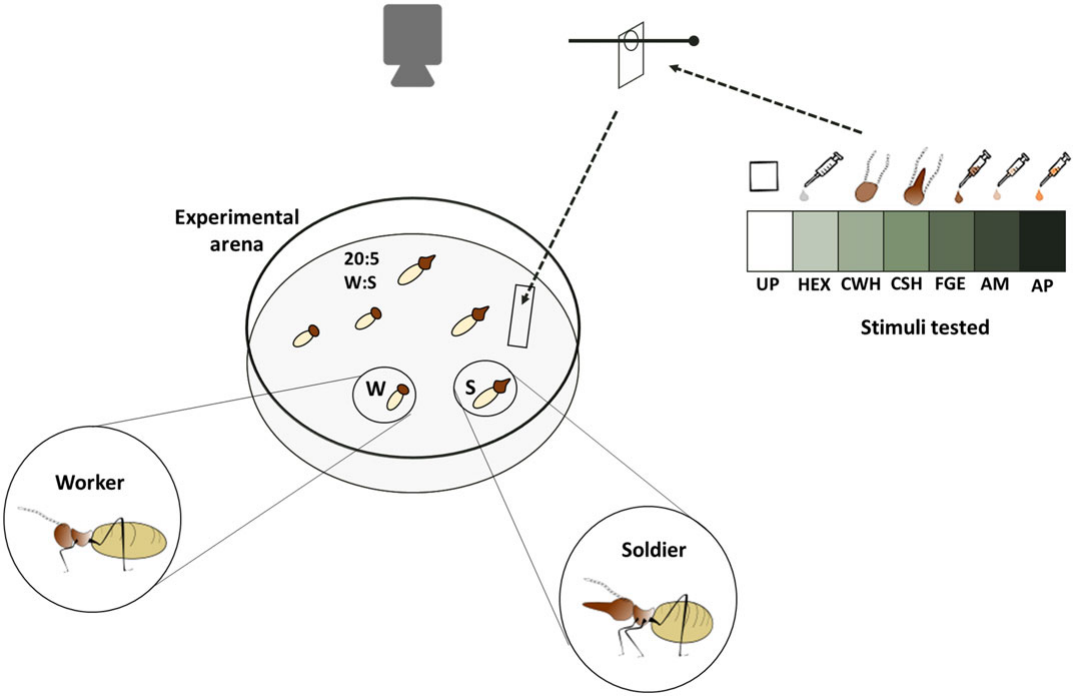
**Experimental settings for the running speed bioassay.** In each experiments *C. cyphergaster* groups composed by 20 workers and 5 soldiers were placed in experimental arenas in which the stimuli were introduced. The tested stimulus was loaded onto a strip of the filter paper (7×3 mm) and introduced immediately into the experimental arena. The stimuli comprised treatments representing an ascending order of alarm pheromone content: from zero pheromones (UP, untreated paper and HEX, hexane) to zero alarm pheromones (CWH, crushed workers head), to increasing purity of alarm pheromones (CSH, crushed soldiers head; FGE, soldier's frontal gland extract; AM, mixture of all monoterpenes found in *C. cyphergaster* frontal gland secretion; AP, alarm pheromone). Experiments were recorded using a reflex camera on a tripod.

Alarm behaviour was bioassayed in the lab aiming to inspect the chemical nature of the alarm observed in the field. To do so, groups of termites (5 soldiers and 20 workers) taken from six field colonies were confined to experimental arenas (Fig. 8) made from plastic Petri dish (ø 85 mm) whose internal bottom surface was lined with moistened filter paper (Whatman no. 1). Each termite group was allowed to acclimatize for at least 2 h prior to the beginning of the assay. The choice of the caste ratio and termite numbers in each arena was a compromise between natural caste proportions (soldier:worker=1:4.5; Cunha et al., 2003) and optimal density to maximize inter-individual contacts and survival (Miramontes and DeSouza, 1996).

Before main experiments, we compared the behavioural responses under red light and dimmed daylight. Although the behavioural response were more pronounced under red light, we had to switch the experimentation to dimmed daylight, once the low-intensity of red light did not allow us to distinguish between workers and soldiers, which are quite similar in terms of body size, shape and coloration (see Mathews, 1977), but we needed to treat them separately.

These bioassays consisted of video-recording, for posterior analysis, the termites reaction when exposed to a given chemical stimulus. Recording was made using digital cameras (Nikon D300s or Canon EOS 500D) fitted with a macro lens. Each recording lasted for 7 min, 2 of which were before the stimulus insertion and 5 after. In doing so, the baseline activity of each group could be debited from its activity after exposure to stimulus, thereby providing inter-group comparable estimates.

Termites’ reaction to stimuli was evaluated quantifying (i) the increment in the number of visually detectable shaking movements in all termites in the test, and (ii) the increment in the running speed of one soldier and one worker arbitrarily selected within the arena at the beginning of the record, i.e. before the stimulus introduction. Running speed was estimated using Mouse-Tracer software, as detailed in Šobotník et al. (2008). Data are presented as the average of the pair of values for soldier and worker. The testing stimulus was loaded onto a piece of filter paper (7×3 mm) which was introduced into the experimental arena through a slit in the dish cap. The filter paper was hung out of reach of termites by a pin bridge over the slit (Fig. 8).

Treatments consisted of two controls, three natural and two synthetic stimuli. Controls consisted of a blank control, presented as untreated filter paper (UP), and a solvent control, presented as filter paper treated with hexane (HEX). Natural stimuli consisted of filter paper impregnated with crushed workers’ heads (CWH), crushed soldiers’ heads (CSH) and extracts of soldiers’ frontal glands (FGE). Synthetic stimuli consisted of filter paper impregnated with commercial standards of monoterpenes found in the frontal gland (see section ‘Chemical identity of alarm components’ above), prepared as a mixture all monoterpenes (AM) and the most active combination of monoterpenes, that is, the alarm pheromone (AP). Crushed head treatments were conducted using workers (CWH) and soldiers (CSH) that had been anesthetized on ice and then dissected into the head and the rest of body. Heads were crushed by tweezers pressure against the piece of a filter paper to release contents of head organs (including the frontal gland reservoir). AM was prepared from monoterpenes standards after quantifying of particular monoterpenes in the mixture of (1S)-α-pinene as an external standard. The standards camphene as well as both enantiomers of α-pinene, β-pinene and limonene were purchased from Sigma-Aldrich; myrcene and (*E*)-β-ocimene originated from IOCB laboratory store. The AP, in ratio observed in soldier head extracts (for details see Table 1) was obtained from preliminary tests with standards of all monoterpenes found in the *C. cyphergaster* frontal gland secretion (see Table 2 for details). Compounds that triggered higher speed of termite motion were selected as the alarm pheromone and used in the main experiments.

These treatments correspond to a sequential increment in the amount of alarm pheromones being tested. The ‘blank control’ and ‘hexane control’ present no compound of termite origin. The ‘workers heads’ treatment provides no alarm pheromone but it provides other typical termite compounds being, hence, an extra ‘control treatment’. The ‘soldier heads’ treatment provides alarm pheromones mixed with other chemicals of termite origin. The ‘frontal glands’ treatment provides mostly alarm pheromones (they are the natural reservoir of this kind of pheromone). The ‘monoterpenes’ treatment provides only alarm pheromones mixed with other monoterpenes, free from termite compounds and, finally, the ‘alarm pheromone’ treatment provides pure alarm pheromone.

In order to confirm dose-dependence of behaviours on alarm pheromones, an extra set of bioassays have been performed using only the synthetic stimuli (AM and AP) now in two distinct doses: 2 gland equivalents and 6 gland equivalents.

### Dose-dependent effects of alarm pheromone on vibroacoustic communication

In addition to shaking behaviour and running movements observed in the video-recordings we further inspected another alarm behaviour, already known for termites (Howse, 1965): drumming, an up and down oscillation of the body in a vertical plane. Because drumming transmits vibration to the substrate, it could be recorded by high sensitivity accelerometers (Brüel & Kjaer type 4507 B 007, nominal sensitivity 100 mW/ms^-2^) attached to the external bottom surface of a Petri dish containing a group of termites (5 soldiers+20 workers, see above). Bioassays were conducted as above, exposing termites to distinct chemical elicitors of either natural or synthetic nature, in the anechoic room at the Faculty of Electrical Engineering (Czech Technical University in Prague, Czech Republic). Recording by Soft dB Tenor recorder (24 bit, sampling frequency 48 kHz) proceeded under red light, on a table hung from the ceiling to prevent undesired vibrations such as background noise or floor vibration. Each Petri dish had its internal bottom surface coarsened to ease termite movements, as lining it with filter paper would damp termite vibrations.

Records were analysed using Matlab software, with post-processing of the signals being based on spectrogram method using windowed Discrete-Time Fourier Transform. An analysis of correspondence between video record and vibratory signals confirmed that the first structural mode of the Petri dish was excited exclusively by the drumming of a termite, and thus only this structural mode was evaluated. The threshold (magenta dash line in Fig. 7A) was based on the effective (root mean square) value computed over all time of recording (120 s before and 300 s after disturbance).

For each recording, a drumming series was defined as a sequence of three consecutive bursts at least one of which was above the threshold. Individual beats combined form a burst. The total number of drumming series observed in a Petri dish along the 300 s of each recording was divided by the total number of individuals (*n*=25) confined there. This number was then subjected to statistical analysis to compare distinct treatments, as described below.

### Statistical analyses

To inspect the effect of stimuli (*x-var1*) on (i) body shaking movements (*y-var*) and (ii) substrate-borne vibratory alarm signals (*y-var*), either in the chemical or vibroacoustic bioassays, data were subjected to generalized linear modelling (GLM) under normal errors. Model simplification was performed by contrast analyses as described with F tests, lumping treatment levels together until a change in deviance with *P*<0.05 was observed, as recommended by Crawley (2007). All analyses were performed in R (R Development Core Team, 2012), followed by residual analysis to check the suitability of the error distribution and model fitting. Analysis for each *y-var* was done separately.

The effect of stimuli (*x-var1*) and caste (*x-var2*) on the ambulatory behaviour of termites (*y-var*) were analysed with linear-mixed effects modelling with ‘stimulus’, ‘caste’ and their first-order interaction as fixed factors, and ‘caste’ nested within ‘nest identity’ as the random factor (Pinheiro and Bates, 2000). The use of this statistical approach was necessary because workers and soldiers (i.e. the categories forming the variable ‘caste’ [*x-var2*]), belong to the same nest and hence are not independent from each other. Likelihood ratio tests compared full models to the null model composed only by random effects.

## References

[BIO014084C1] Adams R. P. (2007). *Identification of Essential Oil Components by Gas Chromatography/Mass Spectrometry* (4th ed.). Ilinois, U.S.A: Allured Books.

[BIO014084C2] Azevedo N. R., Ferri P. H., Seraphin J. C. and Brandão D. (2006). Chemical composition and intraspecific variability of the volatile constituents from the defensive secretion of *Constrictotermes cyphergaster* (Isoptera, Termitidae, Nasutitermitinae). *Sociobiology* 47, 891-902.

[BIO014084C3] Baker R. and Walmsley S. (1982). Soldier defense secretions of the South American termites *Cortaritermes silvestri*, *Nasutitermes* sp N.D. and Nasutitermes kemneri. *Tetrahedron* 38, 1899-1910. 10.1016/0040-4020(82)80039-2

[BIO014084C4] Baker R., Coles H. R., Edwards M., Evans D. A., Howse P. E. and Walmsley S. (1981). Chemical composition of the frontal gland secretion of Syntermes soldiers (Isoptera, Termitidae). *J. Chem. Ecol.* 7, 135-145. 10.1007/BF00988641 24420433

[BIO014084C5] Baker R., Organ A. J., Prout K. and Jones R. (1984). Isolation of a novel triacetoxysecotrinervitane from the termite *Constrictotermes cyphergaster* (Termitidae, sub-family Nasutitermitinae). *Tetrahedron Lett.* 25, 579-580. 10.1016/S0040-4039(00)99943-X

[BIO014084C6] Bartelt R. J., Zilkowski B. W., Cossé A. A., Steelman C. D. and Singh N. (2009). Male-produced aggregation pheromone of the lesser meal-worm beetle *Alphitobius diaperinus*. *J. Chem. Ecol.* 35, 422-434. 10.1007/s10886-009-9611-y 19337774

[BIO014084C7] Bell W. J., Roth L. M. and Nalepa C. A. (2007). *Cockroaches: Ecology, Behavior, and Natural History*, pp. 152-153. Baltimore, USA: The Johns Hopkins University Press.

[BIO014084C76] Blum M. S. (1981). *Chemical defenses of arthropods*. New York: Academic Press.

[BIO014084C8] Blum M. S. (1969). Alarm pheromones. *Annu. Rev. Entomol.* 14, 57-80. 10.1146/annurev.en.14.010169.000421

[BIO014084C9] Blum M. S. (1985). Alarm pheromones. In *Comprehensive Insect Physiology, Biochemistry and Pharmacology* (ed. Kerkut G. A. and Gilbert L. I.), pp. 193-224. New York, USA: Pergamon Press.

[BIO014084C10] Bourguignon T., Šobotník J., Lepoint G., Martin J.-M., Hardy O. J., Dejean A. and Roisin Y. (2011). Feeding ecology and phylogenetic structure of a complex Neotropical termite assemblage, revealed by nitrogen stable isotope ratios. *Ecol. Entomol.* 36, 261-269. 10.1111/j.1365-2311.2011.01265.x

[BIO014084C11] Bourguignon T., Lo N., Cameron S. L., Šobotník J., Hayashi Y., Shigenobu S., Watanabe D., Roisin Y., Miura T. and Evans T. A. (2015). The evolutionary history of termites as inferred from 66 mitochondrial genomes. *Mol. Biol. Evol.* 32, 406-421. 10.1093/molbev/msu308 25389205

[BIO014084C12] Cameron S. L., Lo N., Bourguignon T., Svenson G. J. and Evans T. A. (2012). A mitochondrial genome phylogeny of termites (Blattodea: Termitoidae): robust support for interfamilial relationships and molecular synapomorphies define major clades. *Mol. Phylogenet. Evol.* 65, 163-173. 10.1016/j.ympev.2012.05.034 22683563

[BIO014084C13] Connétable S., Robert A. and Bordereau C. (1998). Role of head-banging in alarm communication in two fungus-growing termites: *Pseudacanthotermes spiniger* and *P*. militaris. *Act. Colloq. Insect. S.* 11, 117-124.

[BIO014084C14] Connétable S., Robert A., Bouffault F. and Bordereau C. (1999). Vibratory alarm signals in two sympatric higher termite species: *Pseudacanthotermes spiniger* and *P*. militaris. *J. Insect. Behav.* 12, 329-342. 10.1023/A:1020887421551

[BIO014084C15] Costa-Leonardo A. M. and Shields K. S. (1990). Morphology of the mandibular glands in workers of *Constrictotermes cyphergaster* (Silvestri) (Isoptera: Termitidae). *Int. J. Insect. Morphol. Embryol.* 19, 61-64. 10.1016/0020-7322(90)90030-S

[BIO014084C77] Crawley M. J. (2007). *The R Book*. London, U.K: John Wiley & Sons.

[BIO014084C16] Cristaldo P. F., DeSouza O. Krasulová J., Jirošová A., Kutalová K., Lima E. R., Šobotník J. and Sillam-Dussès D. (2014). Mutual use of trail-following chemical cues by a termite host and its inquiline. *PLoS ONE* 9, e85315 10.1371/journal.pone.0085315 24465533 PMC3897442

[BIO014084C17] Cunha H. F., Costa D. A., Espírito Santo-Filho K., Silva L. O. and Brandão D. (2003). Relationship between *Constrictotermes cyphergaster* and inquiline termites in the Cerrado (Isoptera: Termitidae). *Sociobiology* 42, 1-10.

[BIO014084C18] Dani F. R., Morgan E. D., Jones G. R., Turillazzi S., Cervo R. and Francke W. (1998). Species-specific volatile substances in the venom sac of hover wasps. *J. Chem. Ecol.* 24, 1091-1104. 10.1023/A:1022358604352

[BIO014084C19] Delattre O., Sillam-Dussès D., Jandák V., Brothánek M., Rücker K., Bourguignon T., Vytisková B., Cvačka J., Jiřiček O. and Šobotník J. (2015). Complex alarm strategy in the most basal termite species. *Behav. Ecol. Sociobiol.* (in press). 10.1007/s00265-015-2007-9

[BIO014084C20] Deligne J., Quennedey A. and Blum M. (1981). The enemies and defence mechanisms of termites. In *Social Insects* (ed. Hermann H.), pp. 1-76. London, UK: Academic Press.

[BIO014084C21] Donovan S. E., Eggleton P. and Bignell D. E. (2001). Gut content analysis and a new feeding group classification of termites. *Ecol. Entomol.* 26, 356-366. 10.1046/j.1365-2311.2001.00342.x

[BIO014084C22] Evans T. A., Lai J. C. S., Toledano E., McDowall L., Rakotonarivo S. and Lenz M. (2005). Termites assess wood size by using vibration signals. *Proc. Natl. Acad. Sci. USA* 102, 3732-3737. 10.1073/pnas.0408649102 15734796 PMC553312

[BIO014084C23] Evans T. A., Inta R., Lai J. C. S. and Lenz M. (2007). Foraging vibration signals attract foragers and identify food size in the drywood termite, *Cryptotermes secundus*. *Insect. Soc.* 54, 374-382. 10.1007/s00040-007-0958-1

[BIO014084C24] Evans T. A., Inta R., Lai J. C. S., Prueger S., Foo N. W., Fu E. W. and Lenz M. (2009). Termites eavesdrop to avoid competitors. *Proc. R. Soc. B. Biol. Sci.* 276, 4035-4041. 10.1098/rspb.2009.1147 PMC2825779 19710058

[BIO014084C25] Goh S. H., Chuah C. H., Tho Y. P. and Prestwich G. D. (1984). Extreme intraspecific chemical variability in soldier defense secretions of allopatric and sympatric colonies of *Longipeditermes longiceps*. *J. Chem. Ecol.* 10, 929-944. 10.1007/BF00987974 24318785

[BIO014084C26] Hager F. A. and Kirchner W. H. (2013). Vibrational and long-distance communication in the termites *Macrotermes natalensis* and *Odontotermes* sp. *J. Exp. Biol.* 216, 3249-3256. 10.1242/jeb.086991 23926309

[BIO014084C27] Hertel H., Hanspach A. and Plarre R. (2011). Differences in alarm responses in drywood and subterranean termites (Isoptera: Kalotermitidae and Rhinotermitidae) to physical stimuli. *J. Insect Behav.* 24, 106-115. 10.1007/s10905-010-9240-x

[BIO014084C28] Hölldobler B. (1999). Multimodal signals in ant communication. *J. Comp. Phisiol. A Sens. Neural Behav. Physiol.* 184, 129-141. 10.1007/s003590050313

[BIO014084C29] Hölldobler B. and Wilson E. O. (2009). *The Superorganism*. London, UK: WW Norton.

[BIO014084C30] Holmgren N. (1909). Termitenstudien: 1. Anatomische Untersuchungen. *K. Sven. Vetenskapsakad. Handl.* 44, 31-215.

[BIO014084C31] Howse P. E. (1962). The perception of vibration by the subgenual organ in *Zootermopsis angusticollis* Emerson and *Periplaneta americana*. *Experientia* 18, 457-458. 10.1007/BF02175857

[BIO014084C32] Howse P. E. (1964). The significance of the sound produced by the termite *Zootermopsis angusticollis* (Hagen). *Anim. Behav.* 12, 284-300. 10.1016/0003-3472(64)90015-6

[BIO014084C33] Howse P. E. (1965). On the significance of certain oscillatory movements of termites. *Insect. Soc.* 12, 335-345. 10.1007/BF02222723

[BIO014084C34] Inta R., Lai J. C. S., Fu E. W. and Evans T. A. (2007). Termites live in a material world: exploration of their ability to differentiate between food sources. *J. R. Soc. Interface* 4, 735-744. 10.1098/rsif.2007.0223 17360255 PMC2373396

[BIO014084C35] Inward D. J. G., Vogler A. P. and Eggleton P. (2007). A comprehensive phylogenetic analysis of termites (Isoptera) illuminates key aspects of their evolutionary biology. *Mol. Phylogenet. Evol.* 44, 953-967. 10.1016/j.ympev.2007.05.014 17625919

[BIO014084C36] Keegans S. J., Billen J., Morgan E. D. and Gökcen O. A. (1993). Volatile glandular secretions of three species of new world army ants, *Eciton burchelli, Labidus coecus*, and *Labidus praedator*. *J. Chem. Ecol.* 19, 2705-2719. 10.1007/BF00980702 24248722

[BIO014084C37] Kirchner W. H., Broecker I. and Tautz J. (1994). Vibrational alarm communication in the dampwood termite *Zootermopsis nevadensis*. *Physiol. Entomol.* 19, 187-190. 10.1111/j.1365-3032.1994.tb01041.x

[BIO014084C38] Krasulová J., Hanus R., Kutalová K., Šobotník J., Sillam-Dussès D., Tichý M. and Valterová I. (2012). Chemistry and anatomy of the frontal gland in soldiers of the Sand termite *Psammotermes hybostoma*. *J. Chem. Ecol.* 38, 557-565. 10.1007/s10886-012-0123-9 22549556

[BIO014084C39] Kutalová K., Bourguignon T., Sillam-Dussès D., Hanus R., Roisin Y. and Šobotník J. (2013). Armed reproductives: evolution of the frontal gland in imagoes of Termitidae. *Arthropod. Struct. Dev.* 42, 339-348. 10.1016/j.asd.2013.04.001 23583752

[BIO014084C40] Leis M., Angelini I., Sbrenna-Micciarelli A. and Sbrenna G. (1994). Further observations on intercaste communication in *Kalotermes flavicollis*: frequence of vibratory movements under different experimental conditions. *Ethol. Ecol. Evol.* 6, 11-16. 10.1080/03949370.1994.10721966

[BIO014084C41] Lo N., Tokuda H., Watanabe H., Rose M., Slaytor K., Maekawa C., Bandi C. and Noda H. (2000). Evidence from multiple gene sequences indicates that termites evolved from wood-feeding cockroaches. *Curr. Biol.* 10, 801-804. 10.1016/S0960-9822(00)00561-3 10898984

[BIO014084C42] Lubin Y. D. and Montgomery G. G. (1981). Defenses of *Nasutitermes* termites (Isoptera, Termitidae) against *Tamandua* anteaters (Edenata, Myrmecophagidae). *Biotropica* 13, 66-76. 10.2307/2387872

[BIO014084C43] Manser M. B. (2001). The acoustic structure of suricates’ alarm calls varies with predator type and the level of response urgency. *Proc. R. Soc. B Biol. Sci.* 268, 2315-2324. 10.1098/rspb.2001.1773 PMC1088882 11703871

[BIO014084C44] Manser M. B. (2013). Semantic communication in vervet monkeys and other animals. *Anim. Behav.* 86, 491-496. 10.1016/j.anbehav.2013.07.006

[BIO014084C45] Mathews A.G.A. (1977). *Studies of termites from Mato Grosso State, Brazil*. Rio de Janeiro, Brazil: Academia Brasileira de Ciências.

[BIO014084C46] Miramontes O. and DeSouza O. (1996). The nonlinear dynamics of survival and social facilitation in *Nasutitermes* termites. *J. Theor. Biol.* 181, 373-380. 10.1006/jtbi.1996.0138

[BIO014084C47] Moura F. M. S., Vasoncellos A., Araújo V. F. P. and Bandeira A. (2006). Feeding habitat of *Constrictotermes cyphergaster* (Isoptera, Termitidae) in an area of Caatinga, Notheast Brazil. *Sociobiology* 48, 373-380.

[BIO014084C48] Noirot C. (1969). Glands and secretions. In *Biology of Termites* (ed. Krishna K. and Weesner F. M.), pp. 89-123. London, UK: Academic Press.

[BIO014084C49] Noirot C. and Quennedey A. (1974). Fine structure of insect epidermal glands. *Annu. Rev. Entomol.* 19, 61-80. 10.1146/annurev.en.19.010174.000425

[BIO014084C50] Perdereau E., Dedeine F., Christidès J.-P. and Bagnères A.-G. (2010). Variations in worker cuticular hydrocarbons and soldier isoprenoid defensive secretions within and among introduced and native populations of the subterranean termite, *Reticulitermes flavipes*. *J. Chem. Ecol.* 36, 1189-1198. 10.1007/s10886-010-9860-9 20859758

[BIO014084C51] Pinheiro J. and Bates D. (2000). *Mixed-effects models in S and S-plus*. Springer-Verlag.

[BIO014084C52] Prestwich G. D. (1984a). Defense mechanisms of termites. *Annu. Rev. Entomol.* 29, 201-232. 10.1146/annurev.en.29.010184.001221

[BIO014084C53] Prestwich G. D. (1984b). Interspecific variation of diterpene composition of *Cubitermes* soldier defense secretions. *J. Chem. Ecol.* 10, 1219-1231. 10.1007/BF00988550 24318907

[BIO014084C54] Quennedey A. (1984). Morphology and ultrastructure of termite defense glands. In *Defensive Mechanisms in Social Insects* (ed. Hermann H. R.), pp. 151-200. New York, USA: Praeger.

[BIO014084C55] Quintana A., Reinhard J., Faure R., Uva P., Bagnéres A.-G., Massiot G. and Clemént J.-L. (2003). Interspecific variation in terpenoid composition of defensive secretions of European *Reticulitermes* termites. *J. Chem. Ecol.* 29, 639-652. 10.1023/A:1022868603108 12757325

[BIO014084C56] R Development Core Team (2012). *R: A Language and Environment for Statistical Computing*. Vienna, Austria: R Foundation for Statistical Computing, http://www.r-project.org/, ISBN: 3-900051-07-0.

[BIO014084C57] Reinhard J. and Clément J.-L. (2002). Alarm reaction of European *Reticulitermes* termites to soldier head capsule volatiles (Isoptera, Rhinotermitidae). *J. Insect Behav.* 15, 95-107. 10.1023/A:1014436313710

[BIO014084C58] Reinhard J., Quintana A., Sreng L. and Clément J. L. (2003). Chemical signals inducing attraction and alarm in European *Reticulitermes* termites (Isoptera, Rhinotermitidae). *Sociobiology* 42, 675-691.

[BIO014084C59] Reynolds E. S. (1963). The use of lead citrate at high pH as an electron-opaque stain in electron microscopy. *J. Cell Biol.* 17, 208-212. 10.1083/jcb.17.1.208 13986422 PMC2106263

[BIO014084C60] Röhrig A., Kirchner W. H. and Leuthold R. H. (1999). Vibrational alarm communication in the African fungus-growing termite genus *Macrotermes* (Isoptera, Termitidae). *Insect Soc.* 46, 71-77. 10.1007/s000400050115

[BIO014084C61] Roisin Y., Everaerts C., Pasteels J. M. and Bonnard O. (1990). Caste-dependent reactions to soldier defensive secretion and Chiral Alarm/Recruitment Pheromone in *Nasutitermes princeps*. *J. Chem. Ecol.* 16, 2865-2875. 10.1007/BF00979479 24263260

[BIO014084C62] Santos C. A. and Costa-Leonardo A. M. (2006). Anatomy of the frontal gland and ultramorphology of the frontal tube in the soldier caste of species of Nasutitermitinae (Isoptera, Termitidae). *Microsc. Res. Techniq.* 69, 913-918. 10.1002/jemt.20365 17029240

[BIO014084C63] Sbrenna G., Micciarelli A. S., Leis M. and Pavan G. (1992). Vibratory movements and sound production in *Kalotermes ﬂavicollis* (Isoptera: Kalotermitidae). In *Biology and Evolution of Social Insects* (ed. Billen J.), pp. 23-238. Leuven, Belgium: Leuven University Press.

[BIO014084C64] Shurin J. B. and Allen E. G. (2001). Effects of competition, predation, and dispersal on species richness at local and regional scales. *Am. Nat.* 158, 624-637. 10.1086/323589 18707356

[BIO014084C65] Šobotník J., Hanus R., Kalinová B., Piskorski R., Cvačka J., Bourguignon T. and Roisin Y. (2008). (*E,E*)-α-Farnesene, an alarm pheromone of the termite *Prorhinotermes canalifrons*. *J. Chem. Ecol.* 34, 478-486. 10.1007/s10886-008-9450-2 18386097

[BIO014084C66] Šobotník J., Jirošová A. and Hanus R. (2010a). Chemical warfare in termites. *J. Insect Physiol.* 56, 1012-1021. 10.1016/j.jinsphys.2010.02.012 20223240

[BIO014084C67] Šobotník J., Bourguignon T., Hanus R., Sillam-Dussès D., Pflegerová J., Weyda F., Kutalová K., Vytisková B. and Roisin Y. (2010b). Not only soldiers have weapons: evolution of the frontal gland in imagoes of the termite families Rhinotermitidae and Serritermitidae. *PLoS ONE* 5, e15761 10.1371/journal.pone.0015761 21209882 PMC3012694

[BIO014084C68] Šobotník J., Sillam-Dussès D., Weyda F., Dejean A., Roisin Y., Hanus R. and Bourguignon T. (2010c). The frontal gland in workers of Neotropical soldierless termites. *Naturwissenschaften* 97, 495-503. 10.1007/s00114-010-0664-0 20352178

[BIO014084C69] Staddon B. W. (1990). Male sternal pheromone glands in Acanthosomatid shield bugs from Britain. *J. Chem. Ecol.* 16, 2195-2201. 10.1007/BF01026930 24264086

[BIO014084C70] Stuart A. M. (1963). Studies of the communication of alarm in the termite *Zootermopsis nevadensis* (Hagen), Isoptera. *Physiol. Zool.* 36, 85-96.

[BIO014084C71] Stuart A. M. (1988). Preliminary studies on the significance of head-banging movements in termites with special reference to *Zootermopsis angusticollis* (Hagen) (Isoptera: Hodotermitidae). *Sociobiology* 14, 49-60.

[BIO014084C72] Valterová I., Křeček J. and Vrkoč J. (1988). Chemical composition of frontal gland secretion in soldiers of *Velocitermes velox* (Isoptera, Termitidae). *Acta Entomol. Bohemos.* 85, 241-248.

[BIO014084C73] Valterová I., Křeček J. and Vrkoč J. (1989). Intraspecific variation in the defence secretions of *Nasutitermes ephratae* soldiers and the biological activity of some of their components. *Biochem. Syst. Ecol.* 17, 327-332. 10.1016/0305-1978(89)90013-6

[BIO014084C74] Vrkoč J., Křeček J. and Hrdý I. (1978). Monoterpenic alarm pheromones in two *Nasutitermes* species. *Acta Entomol. Bohemos.* 75, 1-8.

[BIO014084C75] Yatsynin V. G., Rubanova E. V. and Okhrimenko N. V. (1996). Identification of female-produced sex pheromones and their geographical differences in pheromone gland extract composition from click beetles (Col., Elateridae). *J. Appl. Entomol.* 120, 463-466. 10.1111/j.1439-0418.1996.tb01636.x

